# Colonial Drugs

**Published:** 1910-12-24

**Authors:** 


### Colonial Drugs.

There are many interesting plants in our overseas colonies which demand investigation owing to their presumably active constituents being little known. Professional men in the colonies have their hands too full with private practice to find time or opportunity for conducting research work. Life in the Colonies is crammed too full, as yet, of practical details. The pursuit of money-making is to a very large extent imperative, and the practitioner who, like old Andrew Smith, has a soul above shillings, and who finds

A primrose by the river's brim a wonder of surprising intricacy imperatively urging him to original investigation, has too often no time to occupy himself with the fascinating speculations of science. His colleagues are much in the same case. They possess neither the means nor the technical conveniences furnished by properly equipped and staffed analytical and physiological laboratories to test the therapeutic activities of various new drugs, and it is therefore too much to expect them to embark upon complicated and laborious experiments, which may, after all, yield comparatively little in the way of clinical results. But it is within their power to make inquiries into the reputed virtues of various simples, and to collect specimens for investigation on this side.

This appears to us a business in which the Royal Colonial Institute might legitimately interest itself. It has a large number of non-resident Fellows, many of whom are able botanists and experienced medical men, and an appeal, backed by the influence of the Council of the Institute, may reasonably be expected to encourage them to forward samples and specimens of new drugs and drug-containing plants for investigation here. A collection of such samples would be an incentive to the keen pharmaceutical chemist to engage upon research work with such specimens as seem to contain powerful alkaloidal or glucosidal principles, and it is from such investigation that the most fruitful results are to be expected. We are quite aware that much has already been done in this direction. The pages of our pharmaceutical journals, from time to time, contain interesting accounts of the investigation of this or that plant which in its habitat has a more or less assured reputation as a specific, and it must be confessed that hitherto the results have been discouraging. Cancer specifics, for example, which were freely investigated in Germany at the time when the Emperor Frederic's case aroused world-wide attention, were found to be inert and useless; but there are many, and far less pretentious, claims which need investigation. We need only refer to the various species of geraniaceæ, which are said to be specific in cases of diarrhœa and dysentery—perhaps owing to the relatively high percentage of tannic acid contained in the decoctions—and to the even more interesting East African shrubs whose extracts are said to have the power of killing anthrax germs.

These latter are now being investigated in Germany, and it is in many ways a pity that Dr. Theiler's laboratory in the Transvaal is wholly devoted to veterinary work, else there may be some hope of inducing the workers there to interest themselves in these matters. This is a very wide field for interesting experiment; but what is needed is an organised appeal for material, and a central depot to which samples may be forwarded and where they may be exhibited and obtained by those who desire to work with them. Laymen who know of plants that are reputed simples may do good service by bringing these to the notice of experts. Our readers will doubtless recollect that one of the most powerful drugs in the Pharmacopœia—strophanthus—was discovered in a chance specimen sent home from the Colonies, and there can be little doubt that many other equally interesting combinations are awaiting discovery. If the Colonial Institute cannot deal with the matter, the Kew authorities may perhaps be disposed to undertake the work, or the Pharmaceutical Society may interest itself in the subject.

